# Inhibition of MARK4 Promotes Mitochondrial Biogenesis by Inducing the Phosphorylation of AMPKα to Reduce Myocardial Damage in Rats With Myocardial Infarction

**DOI:** 10.1155/crp/5677597

**Published:** 2025-10-13

**Authors:** Yi Wu, Sai Wang, Jingqi Zhang, Weiyi Wang, Zhi Zeng, Lu Fu, Bin Li

**Affiliations:** ^1^Acute Myocardial Infarction Clinical Research Center, Xianning Central Hospital, 228 Jingui Road, Xianan District, Xianning 437000, Hubei, China; ^2^Laboratory of Cardiovascular Internal Medicine Department, The First Affiliated Hospital of Harbin Medical University, 23 Youzheng Street, Nangang District, Harbin 150001, Heilongjiang, China

**Keywords:** AMP-activated protein kinase α, apoptosis, microtubule affinity–regulating kinase 4, mitochondrial biosynthesis, myocardial infarction

## Abstract

**Purpose:**

Mitochondrial biogenesis is an important factor affecting the development of acute myocardial infarction. MAP/MARK4, a member of the MAP serine/threonine kinase (MARK) family, is involved in a variety of physiological processes. The aim of this study was to investigate the role of microtubule affinity‐regulating kinase 4 (MARK4) in regulating mitochondrial biogenesis in rats with myocardial infarction.

**Methods:**

One week after the left anterior descending, coronary artery was ligated to establish a myocardial infarction model, and MARK4 expression was knocked down in mice. In the fifth week, changes in cardiac function and structure, the myocardial BNP and ATP content, mitochondrial ultrastructure, and the mitochondrial membrane potential and reactive oxygen species levels were observed and detected, and the levels of AMPKα and mitochondrial biogenesis- and apoptosis-related proteins were detected using western blot analysis.

**Results:**

We found that downregulating the expression of MARK4 in rats with myocardial infarction improved cardiac function, alleviated cardiac pathological injury and restored damaged mitochondrial membrane potential, effectively inhibited myocardial apoptosis and restored the myocardial energy supply, and promoted mitochondrial biosynthesis by increasing AMPKα phosphorylation. However, the addition of an AMPKα inhibitor after MARK4 knockdown did not affect mitochondrial biosynthesis in cardiomyocytes, indicating that the inhibition of MARK4 expression may be a promising therapeutic target for myocardial infarction.

**Conclusion:**

Inhibition of MARK4 expression in rats with myocardial infarction plays a cardioprotective role and promotes mitochondrial biogenesis by promoting AMPKα phosphorylation.

## 1. Introduction

With the development of the social economy, acute heart failure caused by myocardial infarction (MI) has become the leading cause of death worldwide. Previous studies of animal and cellular models have shown that the occurrence of myocardial injury after myocardial ischemia is regulated by the interplay of oxidative stress, inflammation, apoptosis, and mitochondrial dysfunction, which contributes to the progression and prognosis of cardiac ischemic disease. Myocardial mitochondrial dysfunction caused by ischemic injury is a key factor leading to cardiac remodeling, pathological hypertrophy, and life-threatening heart failure [1, 2]. Therefore, the search for new therapeutic targets to modulate mitochondrial dysfunction during myocardial injury has received extensive attention. Mitochondria are involved in MI injury by inducing a variety of pathological processes [3–8]. First, most reactive oxygen species (ROS) are produced and released by the mitochondria when the activity of the electron transport chain is reduced. Second, mitochondria act as calcium pumps in cardiomyocytes, and thus when mitochondrial calcium monotransporters are dysregulated, they can cause intracellular calcium overload. Third, mitochondria induce oxidative stress, and cardiomyocyte death initiates an inflammatory response to repair the damaged myocardium. Fourth, although the content of mitochondria in endothelial cells is relatively low, the morphology of mitochondria is disordered during cardiac microvascular injury after MI. As a result, several studies have identified mitochondria as the primary targets of strategies to prevent damage to the heart. Mitochondria are regenerative organelles. Damaged mitochondrial fragments can be metabolized by mitochondrial autophagy and then regenerated by mitochondrial biogenesis [9, 10]. Mitochondrial autophagy determines the rate of degradation of mature mitochondria, whereas mitochondrial biogenesis maintains mitochondrial population turnover [11–13]. The purpose of mitochondrial biogenesis is to rapidly adapt the mitochondria or cardiomyocytes to new energy requirements. Generally, mitochondrial biogenesis is believed to be regulated by mtDNA replication and mitochondria-localized protein synthesis [14]. Studies have shown that the binding of nuclear DNA to transcription factors and posttranscriptional modifications of proteins between the nuclear and mitochondrial genomes are essential for maintaining mitochondrial quality in cardiomyocytes [15]. This process is precisely regulated by peroxisome proliferator–activated receptor-gamma coactivator 1-α (PGC1α) [16]. Therefore, improving mitochondrial biogenesis is considered a promising approach to alleviate myocardial injury [17, 18].

AMP‐activated protein kinase (AMPK) is a heterotrimeric complex composed of three subunits: α, β, and γ. In mammals, the complex includes a combination of two α subunits (α1–α2), two β subunits (β1–β2), and three γ subunits (γ1–γ3). AMPKα has catalytic activity that is promoted by the phosphorylation of Thr172 [19]. AMPKα is closely involved in the pathophysiological processes of various heart diseases. For example, AMPKα overexpression reduces myocardial ischemia−reperfusion injury through the normalization of mitochondrial dynamics and can activate mitochondrial autophagy in a manner dependent on the PINK1/Parkin pathway [20]. AMPKα may also reduce heart failure by inhibiting mitochondria-mediated cardiac remodeling [21]. AMPKα is also an energy receptor of the heart and interferes with energy metabolism in the heart by affecting mitochondria-related glucose metabolism and fatty acid β-oxidation [22]. These findings suggest that AMPKα plays an important role in regulating cardiac mitochondrial function.

MAP/MARK4 is a member of the MAP serine/threonine kinase (MARK) family that participates in a variety of physiological processes, such as inflammation, oxidative stress, apoptosis, and energy homeostasis [23–27]. AKT phosphorylation is increased, AMPK-related kinases are activated, and energy metabolism homeostasis is maintained in the body in MARK4 knockout mice [26]. MARK4 also activates the nuclear factor-κB (NF-κB) pathway by binding to peroxisome proliferator–activated receptor γ (PPARγ) in mouse adipocytes, promoting oxidative stress and inflammation [28, 29]. Recent studies have shown that MARK4 can damage mitochondrial oxidative respiration in porcine placental trophoblast cells, resulting in a decreased ATP content, decreased mitochondrial membrane potential, decreased mitochondrial complex I and III activities, and decreased protein contents of complex I, II, and V subunits. Moreover, an increase in MARK4 expression causes negative changes in mitochondrial biogenesis and structure [30]. Therefore, the inhibition of MARK4 protects mitochondrial function. In addition, MARK4 expression is increased in MI mice, and removing microtubule tyrosination in MARK4 knockout mice can improve cardiac function early after MI [31]. Therefore, MARK4 may be a multifunctional protein involved in mitochondrial function or physiological and pathological processes and can be considered a potential regulator of mitochondrial dysfunction. Our preliminary experiments revealed that decreased MARK4 expression could activate *p*-AMPKα in the heart. However, the regulatory effect of MARK4 on mitochondrial dysfunction after MI is still unclear and deserves further study. Therefore, in the present study, we investigated the effect of MARK4 on mitochondrial function in rats with MI in vivo and in vitro and revealed the exact mechanism of action of MARK4.

## 2. Methodology

### 2.1. Animal Model

Adult male Sprague−Dawley rats (7–8 weeks old, 200–220 g) were purchased from the Experimental Animal Center of the Second Affiliated Hospital of Harbin Medical University and subjected to a 12-h light/dark cycle for 7 days in a temperature- and humidity-controlled environment. All the animal experiments were performed at the First Affiliated Hospital of Harbin Medical University. The procedures of the present study were approved by the Experimental Animal Care and Ethics Committees of the First Affiliated Hospital of Harbin Medical University. Permanent left anterior descending (LAD) coronary artery ligation was performed on the rats, which were ventilated with air using a small animal respirator. Thoracotomy was performed on the left fourth intercostal space, the left ventricle and left atrial appendage were visible, and the LAD coronary artery was fully exposed. The LAD was permanently ligated with 7–0 Prolene sutures. The sutures were passed approximately 2 mm below the tip of the left auricle. Significant color changes and ECG changes in ischemic areas were monitored as indicators of successful coronary occlusion. The thoracotomy was closed with 6–0 Prolene sutures. Sham-operated rats underwent the same procedure without coronary artery ligation. After the recovery of spontaneous breathing, the tracheal tube was removed, and the rats were placed in a warm recovery cage at 37°C until they were fully awake. After the operation, 2.5 × 10^4^ U of penicillin sodium was injected intraperitoneally for 3 consecutive days. After 1 week of feeding, the AAV9 vector carrying a small hairpin RNA (shRNA) for MARK4 under control of the cTnT promoter (AAV9-shMARK4) or the corresponding negative control AAV9-shNC (3 × 10^13^–1 × 10^14^ vp (viral particles) per kg of rat body weight) [32] was administered intravenously to rats with MI. Four weeks after the virus injection, all rats were measured for weight, anaesthetized via the inhalation of isoflurane, and euthanized via carbon dioxide asphyxiation. Afterward, the heart weight and tibia length of the rats were collected and recorded, and the tissues were subsequently collected for analysis.

### 2.2. ECG and Cardiac Echocardiography

A 12-lead electrocardiography system (DECG-03A, Mindray Medical International Co., Ltd., China) was used to conduct an electrocardiographic (ECG) examination immediately after MI. The cardiac function index was measured with a Philips Sonos 5500 multifunction color ultrasound device (Philips, USA) and an 8 MHz transducer index before modeling and four weeks after the virus injection. The left ventricular internal dimension in diastole (LVIDd), left ventricular internal dimension in systole (LVIDs), left ventricular ejection fraction (LVEF), and left ventricular fractional shortening (LVFS) were measured and recorded.

### 2.3. Primary Cardiomyocyte Culture and RNA Interference

Newborn rats were disinfected with 75% alcohol, their hearts were quickly removed, and other tissues were immediately cut and separated. The cut hearts were placed in serum-free DMEM with 1 mL of 0.25% pancreatic enzyme for digestion. After 1 min, the supernatant was placed in DMEM containing 10% serum and 1% cyan/streptomycin. This process was repeated until the samples were thoroughly digested, followed by filtration and centrifugation, and cardiomyocytes were obtained from the supernatant after 1.5 h. Cardiomyocytes were cultured in a 37°C incubator with DMEM plus 10% fetal bovine serum and 5% CO_2_. Cardiomyocytes were exposed to hypoxia in 95% N2 and 5% CO_2_ (12 h, 24 h, or 48 h). Cell viability was calculated via the CCK-8 method, and the appropriate hypoxia duration was selected. For the knock down of MARK4 in cells under the above culture conditions, cells were infected with the lentivirus for MARK4 knockdown at an MOI of 30 for 24 h. After 24 h, the cell medium (serum-free) was replaced with the selection medium (whole medium + 5 μg/mL purinomycin). The transfected cells were preserved until the untransfected cells died. 5 mg of compound C (CpC, BML-275) purchased from MedChemExpress, China, was dissolved in 1.2516 mL of DMSO to prepare a 10 mM stock solution and stored in a −20°C refrigerator. Each time, dilute the original solution to 10 μM/L and add it to the cells for treatment for 24 h to inhibit the expression of AMPKα in the cells.

### 2.4. Western Blot

Total protein extraction was performed using ice-cold RIPA lysis buffer (Beyotime Biotechnology, Shanghai, China) supplemented with 1 × protease inhibitor cocktail (Beyotime, China) and 1 mM phenylmethylsulfonyl fluoride (PMSF). Tissue samples (50 mg) or cell pellets (1 × 10^7^ cells) were homogenized in 500 μL lysis buffer using a Polytron PT 1200 E homogenizer at 4°C, followed by centrifugation at 12,000 × g for 15 min at 4°C to remove insoluble debris. The supernatant protein concentration was quantified using a BCA Protein Assay Kit (Beyotime, China) according to the manufacturer's protocol, with bovine serum albumin (BSA) standards prepared in parallel. Absorbance measurements at 562 nm were performed in triplicate using a SpectraMax M5 microplate reader (Molecular Devices, CA, USA) with background subtraction from reagent blanks. All protein aliquots were stored at −80°C until use, with three independent biological replicates performed for each experimental condition [33].

Antibodies against MARK4 (#4834, 1:1000, CST) and BAX (ab182733, 1:2000, Abcam), BCL-2 (ab196495, 1:1000, Abcam), GAPDH (TA-08, 1:5000, Zsbio), AMPKα (#2532, 1:1000, CST), p-AMPK (AF3423, 1:2000, Affinity), Sirt3 (#5490, 1:1000, CST), TFAM (#7495, 1:1000, CST), and PGC-1*α* (#2178, 1:1000, CST) were used.

### 2.5. Real-Time PCR

Total RNA isolation was performed from approximately 50 mg of myocardial tissue or 1 × 10^6^ cultured cardiomyocytes using the RNAiso Plus reagent (Takara Bio, Shiga, Japan). RNA purity and integrity were verified by NanoDrop 2000 spectrophotometry (A260/A280 ratio > 1.9; Thermo Fisher Scientific). Genomic DNA elimination and reverse transcription were systematically conducted using the PrimeScript RT Reagent Kit with gDNA Eraser (Takara Bio) in a 20 μL reaction system containing 1 μg RNA template. The thermal protocol comprised: 42°C for 2 min (gDNA removal), 37°C for 15 min (reverse transcription), and 85°C for 5 s (enzyme inactivation).

Quantitative PCR analysis was performed using TB Green Premix Ex Taq II (Takara Bio) on a QuantStudio 6 Flex Real-Time PCR System (Applied Biosystems, USA) with the following cycling parameters: initial denaturation at 95°C for 30 s; 45 cycles of 95°C for 5 s, 60°C for 30 s (annealing/extension); followed by melt curve analysis (60°C–95°C, 0.3°C/sec increment). All reactions were conducted in triplicate using 20 μL volumes containing 2 μL cDNA template and 0.2 μM gene-specific primers. The primer sequences were designed using Primer-BLAST (NCBI) and validated for amplification efficiency (90%–110%).

MARK4:

Forward: 5′-TGAAGGGACTCAACCACCC-3′.

Reverse: 5′-TCACCAGGTATAGCGTCTTCTC-3′.

GAPDH:

Forward: 5′-GGCACAGTCAAGGCTGAGAATG-3′.

Reverse: 5′-ATGGTGGTGAAGACGCCAGTA-3′.

Gene expression normalization was performed using GAPDH as the endogenous control, with stability confirmed by geNorm analysis (M value < 0.5). The 2^−ΔΔCT^ method was used to calculate the mRNA levels of each gene [33].

### 2.6. Measurement of the Mitochondrial Membrane Potential

JC-1 staining was used to observe changes in the mitochondrial membrane potential. The ratio of the red/green fluorescence intensity of JC-1 revealed that the number of depolarized mitochondria decreased due to the destruction of red fluorescent J aggregates (Beyotime Biotechnology, China). After the cells were treated as described above, they were washed with PBS, stained with JC-1 according to the instructions, and then observed under a fluorescence microscope (Olympus).

### 2.7. Measurement of Reactive Oxygen Species in Mitochondria

The intracellular ROS levels were measured using a ROS assay kit (Beyotime Biotechnology, China). According to the instructions, the cells were loaded with 10 μM DCFH-DA in serum-free DMEM (pH 7.4) at 37°C, placed in dark conditions for 30 min, then washed with PBS three times to remove the extracellular probe, and then excited at 488 nm under a fluorescence microscope (Olympus). The quantitative fluorescence was measured at an emission wavelength of 525 nm [33]. Alternatively, the sample is observed using CytoFLEX flow cytometry to detect fluorescence signals in the FITC channel for quantitative analysis.

### 2.8. H&E Staining and Masson's Trichrome Staining

After collecting the heart tissue, the specimen was soaked and fixed in 4% paraformaldehyde (0.1 M PBS, pH 7.4) for 48 h at 4°C, stirring continuously. After dehydration with graded ethanol (70%–100%) and removal of xylene, the tissues were infiltrated with paraffin wax under vacuum at 60°C. The slices were then sliced, stained, and finally observed under a microscope (Olympus BX51, Japan) [33].

### 2.9. Transmission Electron Microscopy of Myocardial Tissues

Immediately after the heart tissue was collected, the myocardium was immersed in 4% glutaraldehyde (0.1 M phosphate buffer, pH 7.4) at 4°C for 24 h for initial fixation. After the buffer was thoroughly rinsed, the specimen was placed in 1% osmium tetroxide at room temperature for 2 h, then dehydrated by a graded ethanol series (50%–100%), and embedded in epoxy resin. And then we slice it and we stain it. Finally, the samples were observed by transmission electron microscopy (Hitachi, Japan) [33].

### 2.10. Determination of Myocardial BNP and ATP Levels

Myocardial tissue was collected, homogenized by grinding, and centrifuged, and the resulting supernatant was transferred to an EP tube. The operation was performed in strict accordance with the instructions of the BNP and ATP ELISA kit (China Jianglai Biological).

### 2.11. Detection of Apoptosis

Apoptotic cell populations were quantitatively assessed using a commercial Annexin V-FITC/PI Apoptosis Detection Kit (MULTI SCIENCES, Hangzhou, China) following the manufacturer's protocol. Fluorescence signals were acquired through flow cytometric analysis (BD Accuri C6 Plus Flow Cytometer; BD Biosciences, Shanghai, China) with appropriate compensation settings. Subsequent data processing and apoptotic cell population quantification were performed using FlowJo software (v10.8.1; BD Life Sciences), employing quadrant analysis to differentiate viable (Annexin V-/PI-), early apoptotic (Annexin V+/PI-), late apoptotic (Annexin V+/PI+), and necrotic (Annexin V-/PI+) cell populations. The total percentage of apoptotic cells was determined by performing an Annexin V-FITC/PI assay: Q2 (early apoptotic) + Q3 (late apoptotic).

### 2.12. Statistical Analysis

All the data were statistically analyzed with GraphPad Prism (version 8.0) and are presented as the means ± SDs. Group differences were assessed using either one-way analysis of variance, followed by a Tukey's multiple comparison post hoc test or Student's *t* test for pairwise comparison. Each experiment was repeated at least three times independently. *p* < 0.05 indicated a statistically significant difference.

## 3. Results

### 3.1. The Expression of MARK4 Was Altered During Myocardial Infarction Both In Vivo and In Vitro, and the Knockdown Efficiency of MARK4 and the Duration of Hypoxia in Cardiomyocytes Were Determined

**Figure 1:**
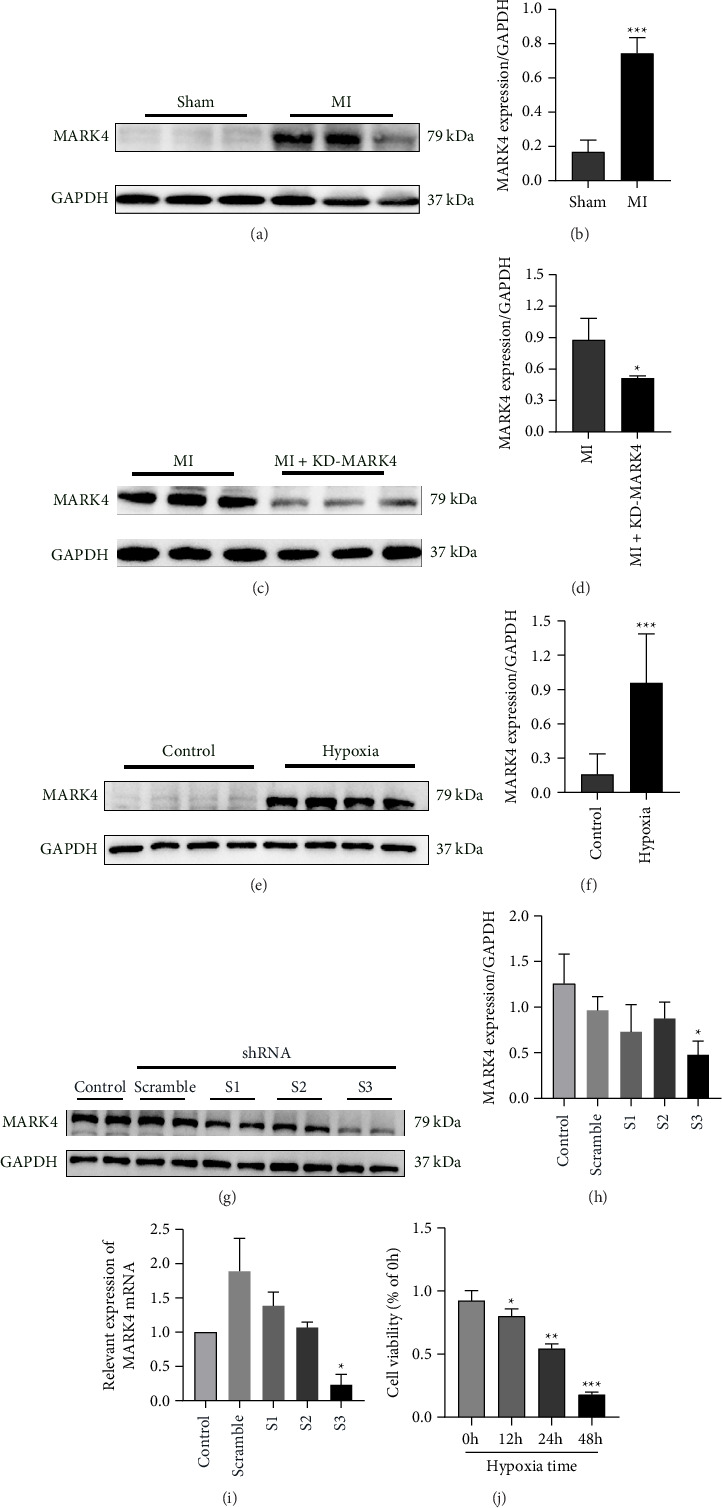
MARK4 was upregulated in vitro and in vivo in response to myocardial infarction. (a, b) MARK4 protein expression in the myocardial tissue of the sham group and MI group. ^∗∗∗^*p* < 0.001 compared with the sham group. (c, d) MARK4 protein expression in the myocardial tissue of the MI group and MI + KD-MARK4 group. ^∗^*p* < 0.05 compared with the MI group. (e, f) MARK4 protein expression in cardiomyocytes from the control group and hypoxia group. ^∗∗∗^*p* < 0.001 compared with the control group. (g–i) Changes in MARK4 protein and mRNA expression in each group; ^∗^*p* < 0.05 compared with the control group. (j) Effects of different durations of hypoxia on cardiomyocyte activity. ^∗^*p* < 0.05, ^∗∗^*p* < 0.01, and ^∗∗∗^*p* < 0.001 compared with 0 h. The data are presented as the means ± SDs (*n* = 3 per group).

The expression of MARK4 was measured by western blotting to investigate the potential role of MARK4 in MI. As shown in Figures 1(a) and 1(b), MARK4 expression was upregulated in rats after MI. As shown in Figures 1(c) and 1(d), 1 week after MI, adeno-associated virus injection through the tail vein successfully knocked down MARK4 in rats. The process of MI was simulated by hypoxia in vitro. Cardiomyocytes were cultured in an anoxic environment for 12, 24, or 48 h, and then the CCK-8 method was used to detect the effects of different durations of hypoxia on cell viability and to select the optimal hypoxia duration. The results of the cell viability test are shown in Figure 1(j). Compared with that of the 0 h hypoxia group, the cell viability of the 12 h hypoxia group was significantly decreased (*p* < 0.05), and the decrease in the cell viability of the 24 and 48 h hypoxia groups was more significant with increasing hypoxia time (*p* < 0.01), indicating that the cardiomyocyte hypoxia model was successfully established in a time-dependent manner. Based on the cell viability value, we finally chose 24 h as the hypoxia treatment time. MARK4 expression was also upregulated after 24 h of hypoxia (Figures 1(e) and 1(f)). Afterward, we constructed a MARK4 shRNA and determined the efficiency of shMARK4 at the protein and mRNA levels. As shown in Figures 1(g), 1(h), and 1(i), compared with those in the control group, the expression levels of the MARK4 protein and mRNA were not significantly lower in the scrambled group (*p* > 0.05). Among the S1, S2, and S3 groups, only the protein and mRNA expression levels of MARK4 in the S3 group were significantly lower than those in the control group (*p* < 0.05). Therefore, we chose the S3 sequence to construct a lentivirus for subsequent cell-based experiments.

### 3.2. Changes in the Electrocardiogram and Cardiac Functional and Structural Parameters in Each Group

**Figure 2:**
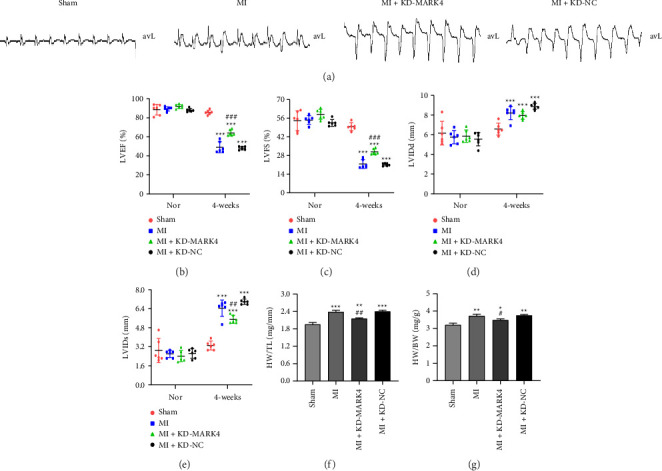
ECG and cardiac function and structural parameters of the rats in each group. (a) Representative ECG of the lead avL of the rats in each group. (b–e) Cardiac parameters of the rats in each group. (f) The ratio of heart weight to body weight. (g) The ratio of heart weight to tibial length. For the analyses at 4 weeks, ^∗∗∗^*p* < 0.001 compared with the sham group; ^##^*p* < 0.01 and ^###^*p* < 0.001 compared with the MI group. The data are presented as the means ± SDs (*n* = 3 per group).

A postoperative electrocardiogram revealed an elevation of the ST segment in lead avL and the formation of a pathological Q wave in the other three groups, except for the sham group, as shown in Figure 2(a). Echocardiography was used to measure the cardiac function and structural parameters of the rats, and no significant differences in the cardiac function parameters were observed among the groups before the operation. As shown in Table 1, after the LAD was ligated into the rats, the virus was injected into the tail vein, and left ventricular function was assessed 4 weeks later. Unlike those in the sham group, the LVIDD and LVIDS significantly increased after 4 weeks in the other three groups, whereas the LVEF and LVFS decreased significantly in the other three groups. As shown in Figures 2(b), 2(c), 2(d), and 2(e), compared with the sham group, the MI group, MI + KD-MARK4 group, and MI + KD-NC group, all presented significantly reduced LVEF and LVFS, whereas LVIDD and LVIDS increased significantly. Compared with the MI group, the MI + KD-MARK4 group presented significant increases in LVEF and LVFS, whereas LVIDD and LVIDS decreased. As shown in Figures 2(f) and 2(g), HW/BW and HW/BL in MI group, MI + KD-MARK4 group, and MI + KD-NC group were increased compared with sham group. Compared with MI group, HW/BW and HW/BL in MI + KD-MARK4 group were significantly decreased. These data indicate that reducing MARK4 expression in rats with MI can increase the LVEF, reduce the left ventricular end diastolic and end systolic diameters, reverse myocardial remodeling, and contribute to the recovery of heart function.

### 3.3. MARK4 Deficiency Can Alleviate Myocardial Fibrosis and Myocardial Injury in Rats With Myocardial Infarction and Improve the Mitochondrial Ultrastructure and Myocardial Energy Supply

**Figure 3:**
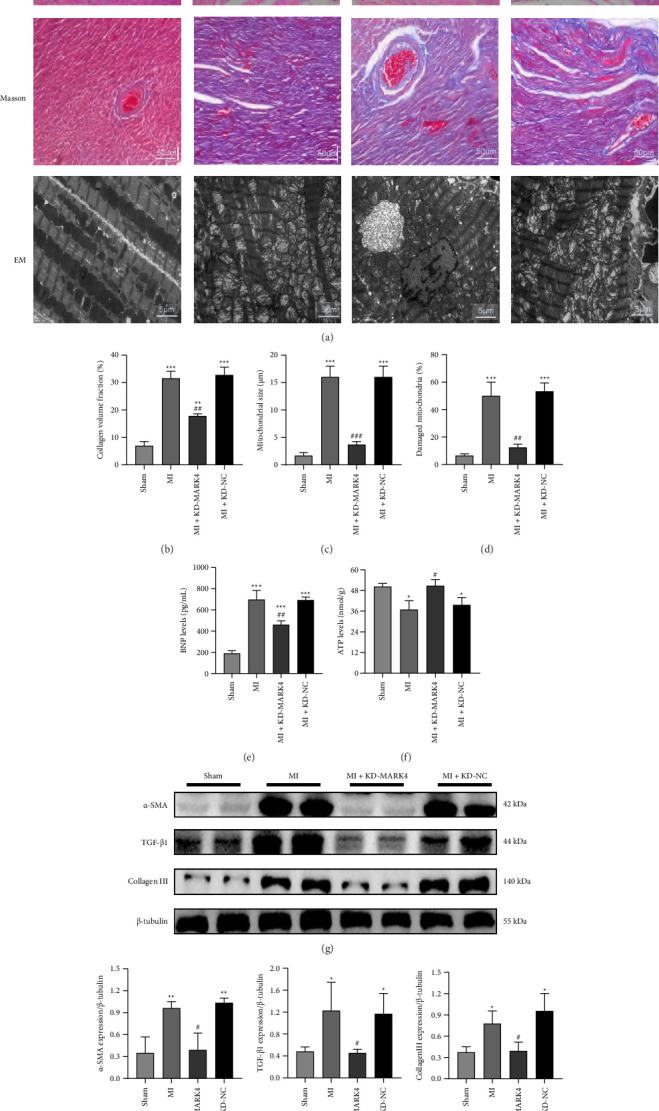
Changes in myocardial tissue structure and fibrosis and the mitochondrial ultrastructure and BNP levels and ATP levels in each group. (a) H&E and Masson's trichrome staining (magnification, × 200). Myocardial transmission electron microscopy images (magnification, × 2000). (b) The ratio of fibrosis area to total area. (c) Mitochondrial size in each group. (d) The ratio of damaged mitochondria to total mitochondria. (e) BNP levels in each group. (f) ATP levels in each group. (g–j) The protein expression of α-SMA, TGF-β1, and Collagen III in each group.^∗^*p* < 0.05 compared with the sham group; ^#^*p* < 0.05 compared with the MI group. The data are presented as the means ± SDs (*n* = 3 per group).

MI characteristics were identified in the left ventricular sections of MI rats via H&E and Masson's trichrome staining (Figures 3(a), 3(b), 3(c), and 3(d)). Compared with those in the sham group, the myocardial morphology of the rats in the MI group, MI + KD-MARK4 group, and MI + KD-NC group was damaged, fibrosis occurred, myocardial fibers were arranged loosely and disorderly with irregular shapes, and myofilament breakage occurred. Compared with those in the MI group, the above morphological changes in the MI + KD-MARK4 group were alleviated. The protein results of fibrosis indicators are also consistent with the above results; that is, compared with the sham group, the expression of α-SMA, TGF-β 1, and Collagen III proteins increased in the MI group. Compared with the MI group, the expression of the above indicators was reduced in the KD-MARK4 group (Figures 3(g), 3(h), 3(i), and 3(j)). This indicates that downregulating MARK4 in rats with MI can alleviate the level of myocardial fibrosis. Myocardial transmission electron microscopy showed that compared with the sham group, rats in the MI group, MI + KD-MARK4 group, and MI + KD-NC group exhibited a disordered arrangement of mitochondrial intimal cristae, swollen mitochondria, and a damaged mitochondrial ultrastructure. Compared with that in the MI group, the number of damaged mitochondria in the MI + KD-MARK4 group was reduced (Figures 3(c) and 3(d)). We used an ELISA to detect the BNP and ATP content in the myocardial tissue from the rats in each group (Figures 3(e) and 3(f)). Compared with that in the sham group, the BNP levels were significantly increased and ATP levels were significantly decreased in the MI group (*p* < 0.05), whereas a significant difference was not observed between the MI + KD-MARK4 group and the sham group. Compared with the MI group, the MI + KD-MARK4 group ATP level was increased and BNP level was decreased, indicating that MARK4 downregulation resulted in reduced myocardial damage and increased energy supply in rats with MI.

### 3.4. MARK4 Deficiency Can Inhibit Myocardial Apoptosis, Reduce the Production of Reactive Oxygen Species, and Restore the Mitochondrial Membrane Potential in Myocardial Infarction Model Rats

**Figure 4:**
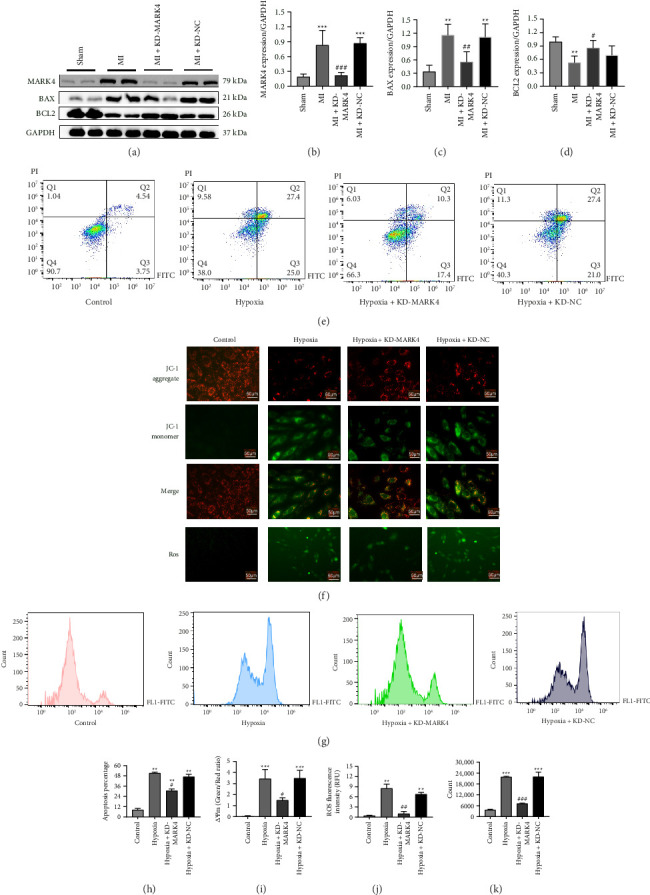
Relationship between MARK4 and myocardial apoptosis. (a–d) The protein expression of MARK4, BAX, and BCL-2 in each group. (e) Flow cytometry was used to detect apoptosis in cardiomyocytes from each group. (f) Observation of the mitochondrial membrane potential (magnification, × 200) and the mitochondrial reactive oxygen level (magnification, × 200) in cardiomyocytes from each group. (g) Flow cytometry was used to detect reactive oxygen species in cardiomyocytes from each group. (h) The ratio of apoptotic cardiomyocytes to total cardiomyocytes. (i) Statistical analysis results of mitochondrial membrane potential in each group. (j) Statistical analysis of reactive oxygen species in each group. (k) Statistical flow analysis of reactive oxygen species in each group. ^∗^*p* < 0.05, ^∗∗^*p* < 0.01, and ^∗∗∗^*p* < 0.001 compared with the sham group or control group; ^#^*p* < 0.05 and ^##^*p* < 0.01 compared with the MI group or hypoxia group. The data are presented as the means ± SDs (*n* = 3 per group).

ROS production and the mitochondrial membrane potential were detected in tissues and cells from rats with MI and decreased MARK4 expression in vivo and in vitro to investigate the effects of MARK4 on cardiomyocyte apoptosis. The levels of apoptosis-related proteins, the apoptosis rate, and the fluorescence intensity of ROS were detected via flow cytometry, and the mitochondrial membrane potential and ROS production were detected via a fluorescent dye. Compared with that in the sham group, the expression of the apoptotic protein BAX was increased and the expression of the antiapoptotic protein BCL2 was decreased in the MI group (*p* < 0.01). However, compared with the MI group, the MI + KD-MARK4 group presented a decrease in in the expression of BAX and an increase in BCL2 expression, and a significant difference was not observed between the MI + KD-MARK4 group and the sham group (Figures 4(a), 4(b), 4(c), and 4(d)). Compared with those of the control group, the apoptosis rates of the hypoxia group, hypoxia + KD-MARK4 group, and hypoxia + KD-NC group increased (*p* < 0.01), but compared with those of the hypoxia group, the apoptosis rates of the hypoxia + KD-MARK4 group were significantly decreased (Figures 4(e) and 4(h)). We subsequently measured the mitochondrial membrane potential and ROS production in hypoxic cardiomyocytes. Compared with those in the control group, the mitochondrial membrane potential levels in the hypoxia group and the hypoxia + KD-MARK4 group were decreased, and the production of ROS was increased. However, compared with the hypoxia group, these changes were reversed in the hypoxia + KD-MARK4 group (Figures 4(f), 4(g), 4(i), 4(j), and 4(k)). Overall, the above results suggest that MARK4 can regulate myocardial apoptosis, the mitochondrial membrane potential, and ROS production, indicating that MARK4 deficiency can reduce myocardial apoptosis and ROS production and reverse the decrease in the mitochondrial membrane potential in rats with MI and hypoxic cells.

### 3.5. Effects of MARK4 Deficiency on AMPKα Phosphorylation and Mitochondrial Biogenesis

**Figure 5:**
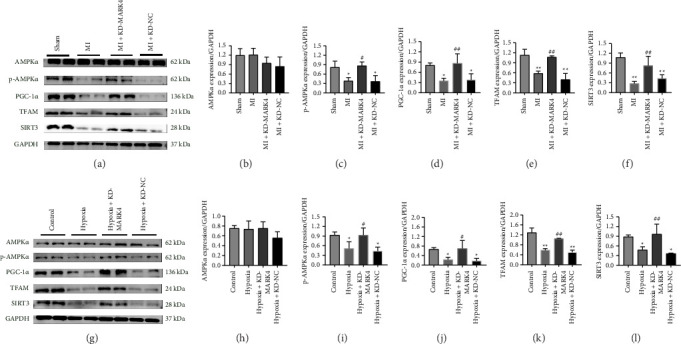
Relationship between MARK4 and mitochondrial biogenesis. (a–f) The protein levels of AMPKα, p-AMPKα, PGC-1α, TFAM, and SIRT3 in the myocardial tissues from the sham group, MI group, MI + KD-MARK4 group, and MI + KD-NC group. (g–l) The protein levels of AMPKα, p AMPKα, PGC-1α, TFAM, and SIRT3 in cardiomyocytes from the control group, hypoxia group, hypoxia + KD-MARK4 group, and hypoxia + KD-NC group. ^∗^*p* < 0.05 and ^∗∗^*p* < 0.01 compared with the sham group or control group; ^#^*p* < 0.05 and ^##^*p* < 0.01 compared with the MI group or hypoxia group. The data are presented as the means ± SDs (*n* = 3 per group).

We interfered with MARK4 expression in vivo and in vitro to elucidate the effect of MARK4 deficiency on mitochondrial biogenesis in rats with MI. First, AMPKα and p-AMPKα levels were detected, and the inhibition of MARK4 expression did not affect the expression of AMPKα but increased the level of p-AMPKα. The results of the in vivo and in vitro experiments were consistent. Moreover, the expression of the key factors of mitochondrial biogenesis, PGC-1α, TFAM, and SIRT3, was decreased in myocardial infarct tissues and hypoxic cardiomyocytes, and interference with MARK4 expression reversed the decrease in the expression of these key factors of mitochondrial biogenesis (Figures 5(a), 5(b), 5(c), 5(d), 5(e), 5(f), 5(g), 5(h), 5(i), 5(j), 5(k), and 5(l)). These results suggest that a reduction in MARK4 expression can promote AMPKα phosphorylation and mitochondrial biogenesis in rats with MI and hypoxic cardiomyocytes.

### 3.6. MARK4 Deficiency Promotes Mitochondrial Biogenesis in Cardiomyocytes by Increasing AMPKα Phosphorylation

**Figure 6:**
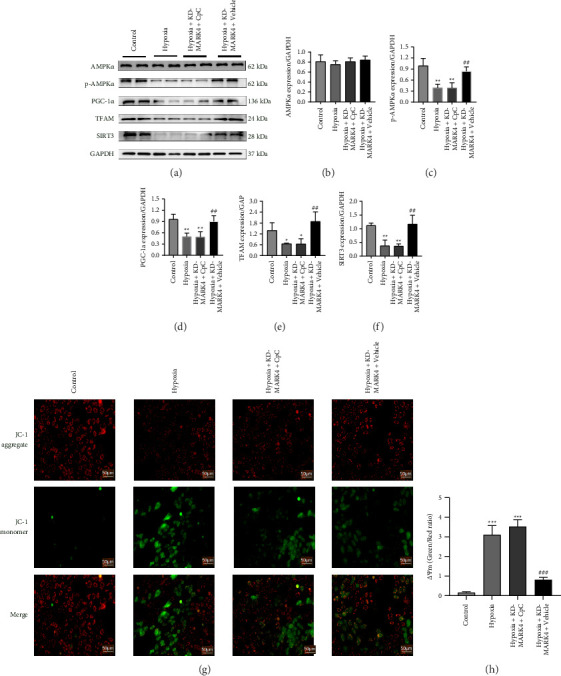
Relationships among MARK4, AMPKα phosphorylation, and mitochondrial biogenesis. (a–f) The protein levels of AMPKα, p-AMPKα, PGC-1α, TFAM, and SIRT3 in cardiomyocytes from the control group, hypoxia group, hypoxia + KD-MARK4+CpC group, and hypoxia + KD-MARK4+vehicle group. (g) Observation of the mitochondrial membrane potential (magnification, × 200) in cardiomyocytes from each group. (h) Statistical analysis results of mitochondrial membrane potential in each group. ^∗^*p* < 0.05 and ^∗∗^*p* < 0.01 compared with the control group. ^#^*p* < 0.05 and ^##^*p* < 0.01 compared with the hypoxia group. The data are presented as the means ± SDs (*n* = 3 per group).

We knocked down MARK4 in cardiomyocytes after 24 h of hypoxia and added the common AMPKα inhibitor CpC to observe the effect of the inhibition of AMPKα phosphorylation on mitochondrial biogenesis and to clearly demonstrate that interference with MARK4 expression affects mitochondrial biogenesis through AMPKα-dependent phosphorylation. Compared with those in the control group, the expression levels of p-AMPKα, PGC-1α, TFAM, and SIRT3 in the hypoxia group and hypoxia + KD-MARK4+CpC group were significantly decreased (*p* < 0.01). However, compared with the hypoxia group, hypoxia + KD-MARK4 reversed the reduction in these factors, while a significant difference was not observed between the hypoxia + KD-MARK4 + CpC group and the hypoxia group. The administration of the AMPKα inhibitor CpC to the hypoxia + KD-MARK4 group reversed the promotion of mitochondrial biogenesis. These results suggest that interfering with MARK4 expression can promote mitochondrial biogenesis in cardiomyocytes by increasing AMPKα phosphorylation (Figures 6(a), 6(b), 6(c), 6(d), 6(e), and 6(f)).We then examined the mitochondrial membrane potentials of cardiomyocytes in each group. Compared with the control group, the mitochondrial membrane potential levels in the hypoxia group and the hypoxia + KD-MARK4 + CpC group were decreased. However, compared with the hypoxia group, the hypoxia + KD-MARK4 + Vehicle group was able to reverse these membrane potential changes (Figures 6(g) and 6(h)).

## 4. Discussion

Acute myocardial infarction (AMI) is a common cardiovascular disease encountered in the clinic, with a high hospitalization rate and high mortality rate, which places a heavy burden on the social economy [34]. As important organelles in cardiomyocytes, mitochondria not only participate in cellular energy production but also play important roles in regulating intracellular ROS production, intracellular ion homeostasis, and signal transduction. Mitochondrial dysfunction is associated with a variety of cardiovascular diseases [35]. Studies have shown that mitochondrial function includes mitochondrial dynamics, mitochondrial biogenesis, and mitochondrial autophagy. The purpose of mitochondrial biogenesis is to rapidly adapt the mitochondria or cardiomyocytes to new energy requirements. Therefore, improving mitochondrial biogenesis is considered a promising approach for alleviating ischemic heart disease [17, 18]. This finding was also confirmed in our current study, in which the mitochondrial structure of rats with MI is disrupted, myocardial apoptosis and ROS production are increased, ATP levels and mitochondrial biogenesis are decreased, and the mitochondrial membrane potential is decreased, which is consistent with the results of other studies [36–40]. In this study, we demonstrated that the inhibition of MARK4 expression in vivo can alleviate myocardial apoptosis, cardiac fibrosis, and mitochondrial ultrastructural damage caused by ischemia and hypoxia; increase mitochondrial biogenesis; and increase the cardiac energy supply. These cardioprotective effects play a vital role by activating AMPKα phosphorylation to increase mitochondrial biosynthesis. The improvement in mitochondrial biogenesis is eliminated after AMPKα inhibition.

The exact mechanism by which MARK4 expression inhibition exerts cardioprotective effects remains elusive. Microtubule affinity‐regulating kinase 4 (MARK4), together with MARK3, MARK2, and MARK1, constitute the microtubule affinity regulatory kinase protein family [41]. The human MARK4 gene is located on the long arm of chromosome 19 and is 53,992 bp long. MARK4 kinase is a 752 amino acid protein that is highly evolutionarily conserved, suggesting that MARK4 has an important biological function [30]. The primary biological function of MARK4 is to phosphorylate microtubule-associated proteins, thereby increasing the number of dynamic microtubules and promoting cell division, cell shape changes, cell cycle control, and cell polarity determination [42]. Previous studies have shown that MARK4 induces oxidative stress and mitochondrial dysfunction through the IKKα/NF-κB signaling pathway in porcine placental trophoblast cells [30]. These data establish a novel regulatory role for MARK4 in mitochondrial function. In our study, we showed for the first time that the levels of MARK4 in myocardial tissue and hypoxic cardiomyocytes were significantly higher than those in the sham operation and control groups at 1 month after MI, and after administering an adeno-associated virus to inhibit MARK4 expression, we observed improvements in the structure and function of the rat heart, similar to the results previously published by Yu et al. [31]. A previous study published in Nature showed that MARK4-deficient mice (MARK4^−/−^) had a significant higher LVEF in the first week after the ligation of the LAD branch coronary artery [31], and in this study, we still observed an improvement in the ejection fraction 1 month later. We also observed the significant inhibition of myocardial apoptosis, improvement in the mitochondrial ultrastructure, recovery of the damaged mitochondrial membrane potential, and increased mitochondrial biogenesis.

AMPKα is widely recognized as a sensor of the cellular energy state, regulating energy metabolic processes under physiological and pathological conditions. In addition, AMPK acts as a key controller of cellular homeostasis and plays a key role in cardiovascular disease, diabetes, and cancer. AMPKα is highly expressed in the heart, and an increasing number of reports have linked AMPKα with myocardial ischemia. Loss of AMPKα activity reportedly contributes to ischemic heart injury [42]. In both brain and heart ischemic injury, inhibition of AMPK/PGC-1α activation can reduce mitochondrial biosynthesis, thereby aggravating cardiac and brain ischemic injury and suggesting that mitochondrial biosynthesis is regulated by AMPKα [43, 44]. Therefore, for the first time, we investigated the mechanism by which reduced MARK4 expression improves myocardial ischemic injury, which may be related to the activation of AMPKα. Our data clearly show that the inhibition of MARK4 stimulates AMPKα activation and enhances mitochondrial biosynthesis. Moreover, treatment with increasing concentrations of AMPKα inhibitors further demonstrated that the inhibition of MARK4 did not enhance mitochondrial biogenesis, confirming that AMPKα plays a key role in inhibiting the promotion of mitochondrial biogenesis by MARK4 in the ischemic myocardium.

In conclusion, the inhibition of MARK4 expression in rats after MI can alleviate myocardial injury, improve cardiac function, inhibit cardiomyocyte apoptosis, and promote mitochondrial biogenesis by increasing AMPKα phosphorylation. This study provides an experimental basis for the application of MARK4 in the treatment of AMI, and future studies elucidating the potential clinical application of MARK4 will be very interesting.

## Figures and Tables

**Table 1:** Effect of MARK4 knockdown on cardiac function in MI model rats (means ± SDs, *n* = 6).

Parameters	Sham		MI		MI + KD-MARK4		MI + KD-NC	
	Nor1	4 weeks	Nor2	4 weeks	Nor3	4 weeks	Nor4	4 weeks
LVEF (%)	88.50 ± 5.61	85.60 ± 2.38	89.38 ± 2.18	49.00 ± 5.90^^ ^ ^^	91.98 ± 2.21	64.05 ± 3.14^&&&^	88.02 ± 1.77	48.10 ± 1.78^$$$^
LVFS (%)	54.17 ± 7.40	49.70 ± 2.92	54.65 ± 3.24	21.65 ± 3.30^^ ^ ^^	58.93 ± 3.76	30.78 ± 2.16^&&&^	52.53 ± 2.56	21.22 ± 1.01^$$$^
LVIDd (mm)	6.16 ± 1.20	6.57 ± 0.61	5.75 ± 0.68	8.21 ± 0.68^^ ^ ^^	5.85 ± 0.62	7.95 ± 0.38^&&&^	5.55 ± 0.68	8.87 ± 0.30^$$$^
LVIDs (mm)	2.88 ± 1.01	3.31 ± 0.38	2.60 ± 0.30	6.44 ± 0.69^^ ^ ^^	2.42 ± 0.47	5.50 ± 0.33^&&&^	2.64 ± 0.40	6.99 ± 0.23^$$$^

*Note:* In the MI group, ^^  ^  ^^*p* < 0.001 compared with Nor2; in the MI + KD-MARK4 group, ^&&&^*p* < 0.001 compared with Nor3; and in the MI + KD-NC group, ^$$$^*p* < 0.001 compared with Nor4.

## Data Availability

The data that support the findings of this study are available from the corresponding author upon reasonable request.

## Nomenclature

MI: Myocardial infarction
MARK4: Microtubule affinity‐regulating kinase 4
ROS: Reactive oxygen species
LVIDd: Left ventricular internal dimension in diastole
LVIDs: Left ventricular internal dimension in systole
LVEF: Left ventricular ejection fraction
LVFS: Left ventricular fractional shortening
